# Loss of Hepatocyte FOXA3 Improves MASH and Atherosclerosis in Hyperlipidemic Ldlr-Deficient Mice

**DOI:** 10.3390/ijms27031468

**Published:** 2026-02-02

**Authors:** Hui Wang, Shuwei Hu, Jiayou Wang, Raja Gopoju, Li Lin, Lakshitha Gunawardana, Xinwen Wang, Liya Yin, Yanqiao Zhang

**Affiliations:** 1 Department of Internal Medicine and the Translational Cardiovascular Research Center, University of Arizona College of Medicine—Phoenix, Phoenix, AZ 85004, USAjwang11@arizona.edu (J.W.); rajag@arizona.edu (R.G.);; 2 School of Biomedical Sciences, Kent State University, Kent, OH 44272, USA; 3 Department of Pharmaceutical Sciences, Northeast Ohio Medical University, Rootstown, OH 44272, USA

**Keywords:** MASLD, atherosclerosis, lipogenesis, liver, bile acids

## Abstract

Forkhead box protein A3 (FOXA3), also known as hepatocyte nuclear factor 3g (HNF3g), is a member of the FOX family of transcription factors and regulates lipid and glucose metabolism and liver regeneration. Hepatic FOXA3 is reduced in obesity and patients with metabolic dysfunction-associated steatohepatitis (MASH). So far, it remains unknown whether hepatic FOXA3 is essential for regulating lipid metabolism or metabolic dysfunction-associated liver disease (MASLD). In this study, we first investigated whether genetic inactivation of hepatocyte *Foxa3* affected the development of MASLD/MASH in C57BL/6 mice and then explored whether loss of hepatocyte *Foxa3* regulated atherosclerosis development in *Ldlr*-deficient mice. Inactivation of *Foxa3* in hepatocytes did not affect the development of Western diet-induced MASLD/MASH in C57BL/6 mice but attenuated MASH development in Western diet-fed *Ldlr*-deficient mice. Moreover, genetic loss of hepatocyte *Foxa3* ameliorated hyperlipidemia and atherosclerosis in *Ldlr*-deficient mice. In *Ldlr*-deficient mice, loss of hepatocyte *Foxa3* resulted in reduced expression of lipogenic, pro-inflammatory, or fibrogenic genes in the liver and reduced cholic acid levels in plasma and bile. Thus, hepatocyte FOXA3 loss confers protection against the development of MASH and atherosclerosis in hyperlipidemic *Ldlr*-deficient mice.

## 1. Introduction

Metabolic dysfunction-associated steatotic liver disease (MASLD), formerly referred to as nonalcoholic fatty liver disease (NAFLD), is a common chronic liver disease worldwide, with a global prevalence rate of 30% [1]. Metabolic dysfunction-associated steatohepatitis (MASH) is the progressive form of MASLD. MASH may further progress to cirrhosis and hepatocellular carcinoma (HCC). MASLD/MASH is often associated with obesity and diabetes, and increases the likelihood of type 2 diabetes, cardiovascular disease, and extrahepatic cancer [2]. Although the Food and Drug Administration (FDA) has approved Resmetirom (Rezdiffra), a thyroid hormone receptor β agonist, and Wegovy (semaglutide), a GLP-1 receptor agonist for weight loss, for the treatment of MASH with fibrosis, their efficacy remains limited compared to placebo [3,4].

Forkhead box protein A3 (FOXA3), also known as hepatocyte nuclear factor 3g (HNF3g), is a member of the forkhead box (FOX) family of transcription factors. FOXA3 plays a significant regulatory role during development and adult tissue homeostasis. It regulates gene expression by binding to specific DNA elements in target genes or by functioning as a “pioneer factor,” binding to compacted chromatin and allowing access by other transcription factors. FOXA3 is highly expressed in the liver, with undetectable levels in many different organs, including the lung, heart, adipose tissue, and spleen [5]. FOXA3 is required for maintaining glucose homeostasis during a prolonged fast [6]. Hepatic FOXA3 is reduced in high-fat diet-induced obese or genetically obese mice and MASH patients [7]. Overexpression of human FOXA3 in the liver has been shown to protect against atherosclerosis by regulating ApoA-I and HDL metabolism (7) and to prevent Western diet-induced obesity and MASH via Takeda G protein-coupled receptor 5 (TGR5) [8]. Hepatocyte-specific FOXA3 overexpression also accelerates hepatocyte proliferation and attenuates liver injury in a CCl_4_-induced acute liver injury model, whereas global inactivation of FOXA3 impairs liver regeneration after partial hepatectomy [9]. In addition, adenovirus-induced FOXA3 overexpression is shown to prevent bile duct ligation-induced liver fibrosis in rats [10,11]. All these gain-of-function studies have demonstrated that hepatic FOXA3 may protect against diet- or toxin-induced liver injury or steatohepatitis.

Global inactivation of *Foxa3* does not cause morphological defects but reduces the expression of several hepatocyte-specific genes (e.g., phosphoenolpyruvate carboxykinase, transferrin) by 50–70% in mice [12]. To date, the role of hepatocyte-specific FOXA3 loss in MASLD/MASH or atherosclerosis remains unknown. Here, we show that genetic inactivation of *Foxa3* in hepatocytes does not affect the development of MASLD/MASH in Western diet-fed C57BL/6 mice. Unexpectedly, genetic loss of hepatocyte *Foxa3* in *Ldlr*-deficient mice attenuates Western diet-induced MASLD/MASH, hyperlipidemia, and atherosclerosis. We further show that hepatocyte-specific loss of *Foxa3* in these hyperlipidemic mice reduces hepatic expression of lipogenic, pro-inflammatory, and fibrogenic genes, as well as cholic acid levels. Our data suggest that loss of hepatocyte FOXA3 may prevent the development of MASH and atherosclerosis in severely hyperlipidemic mice.

## 2. Results

### 2.1. Genetic Loss of Hepatocyte Foxa3 Does Not Affect Hfcf Diet or Western Diet-Induced Masld

**Figure 1:**
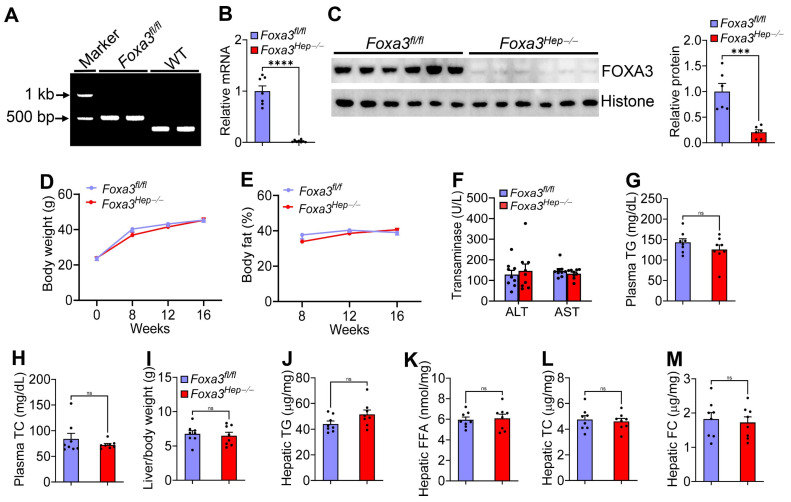
**Genetic loss of hepatocyte FOXA3 in C57BL/6 mice has no impact on lipid homeostasis or MASLD.** (**A**) PCR genotyping of floxed *Foxa3* mice. The band size for floxed mice and wild-type mice was 510 bp and 372 bp, respectively. (**B**–**M**) *Foxa3^fl/fl^* mice and hepatocyte-specific *Foxa3*^−/−^ (*Foxa3^Hep−/−^*) mice were fed a high-fat/cholesterol/fructose (HFCF) diet for 16 weeks (*n* = 8 per group). Hepatic *Foxa3* mRNA (**B**) and protein (**C**) levels were analyzed. Body weight (**D**) and body fat content (**E**) were measured. Plasma levels of ALT and AST (**F**), TG (**G**), and TC (**H**) were examined. The liver-to-body weight ratio (**I**) and hepatic levels of TG (**J**), FFA (**K**), TC (**L**), and FC (**M**) were determined. Data are expressed as mean ± SEM. ns, not significant. *** *p* < 0.001, **** *p* < 0.0001.

Previous studies have shown that hepatic FOXA3 is reduced in MASH patients and obese mice [7], and that overexpression of hepatic FOXA3 protects against atherosclerosis in *Apoe*^−/−^ mice [7] and prevents Western diet-induced obesity and MASH [8]. So far, it remains unknown whether genetic loss of hepatic FOXA3 affects the development of atherosclerosis, obesity, or MASH. To address this question, we generated floxed *Foxa3* (*Foxa3^fl/fl^*) mice with loxP sites flanking exon 2 (Figure 1A). We then i.v. injected *Foxa3^fl/fl^* mice with AAV8-TBG-Null or AAV8-TBG-Cre to generate control (*Foxa3^fl/fl^*) mice or hepatocyte-specific *Foxa3*^−/−^ (*Foxa3^Hep−/−^*) mice. Subsequently, the mice were fed a diet enriched in high-fat/cholesterol/fructose (HFCF) for 16 weeks.

Examination of hepatic *Foxa3* expression shows that hepatic *Foxa3* mRNA levels were reduced by ~98% (Figure 1B) and FOXA3 protein levels were decreased by 80% (Figure 1C). There were no changes in body weight increase (Figure 1D), body fat content (Figure 1E), plasma levels of ALT or AST (Figure 1F), or triglyceride (TG) (Figure 1G) or total cholesterol (TC) (Figure 1H). The ratio of the liver to body weight did not change (Figure 1I). In addition, loss of hepatocyte *Foxa3* did not affect hepatic levels of TG (Figure 1J), free fatty acids (FFAs) (Figure 1K), TC (Figure 1L), or free cholesterol (FC) (Figure 1M).

In a separate study, *Foxa3^fl/fl^* mice and *Foxa3^Hep−/−^* mice were fed a Western diet containing 42% fat/0.2% cholesterol for 16 weeks. There was no change in energy expenditure (Supplementary Figure S1A) or glucose tolerance (Supplementary Figure S1B). Similar to what we have observed in mice fed an HFCF diet, genetic ablation of hepatocyte *Foxa3* did not affect plasma levels of TG or TC, or hepatic levels of TG, FFA, TC, or FC (Supplementary Figure S1C–H).

Taken together, our data suggest that loss of hepatocyte FOXA3 does not affect HFCF diet or Western diet-induced MASLD or plasma lipid levels.

### 2.2. Loss of Hepatocyte Foxa3 Reduces the Bile Acid Pool Size in Hfcf Diet-Fed Mice

**Figure 2:**
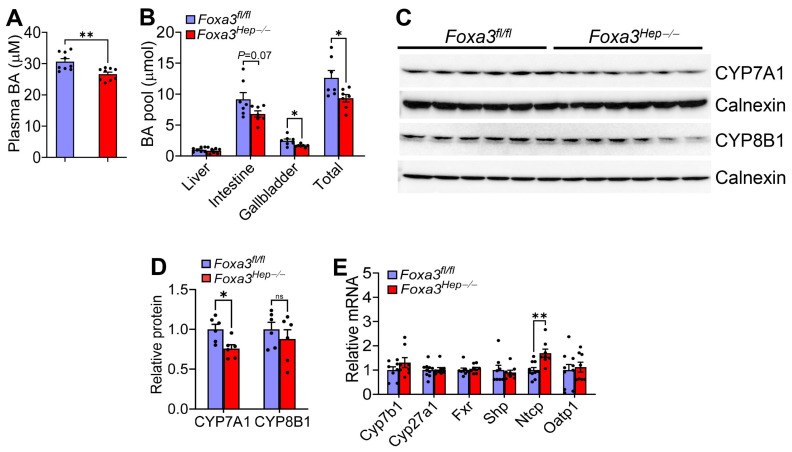
**Genetic loss of hepatocyte FOXA3 in C57BL/6 mice lowers plasma bile acid levels. ***Foxa3^fl/fl^* mice and *Foxa3^Hep−/−^* mice were fed an HFCF diet for 16 weeks (n = 8 per group). (**A**) Plasma bile acid levels. (**B**) Bile acid pool size. (**C**,**D**) Hepatic protein levels. (**E**) Hepatic mRNA levels of genes involved in bile acid metabolism were determined. Data are expressed as mean ± SEM. ns, not significant. * *p* < 0.05, ** *p* < 0.01.

We previously showed that overexpression of hepatic FOXA3 regulates bile acid metabolism [8]. So far, the effect of genetic loss of hepatocyte FOXA3 on bile acid metabolism has not been investigated. *Foxa3^Hep−/−^* mice had reduced bile acid levels in the plasma (Figure 2A) and the gallbladder (Figure 2B), and reduced bile acid pool size (Figure 2B). In the liver, cholesterol 7α-hydroxylase (CYP7A1) protein levels were significantly reduced, whereas sterol 12α-hydroxylase (CYP8B1) protein levels were unchanged (Figure 2C,D). In addition, hepatic level of Na-taurocholate co-transporting polypeptide (*Ntcp*), which transports bile salts from circulation to the liver, was significantly induced, which may account for reduced plasma bile acid levels. In contrast, hepatic mRNA levels of *Cyp7b1*, *Cyp27a1*, farnesoid X receptor (*Fxr*), small heterodimer partner (*Shp*), or organic anion transporter 1 (*Oatp1*) were unchanged (Figure 2E). Nonetheless, the change in bile acid homeostasis appears insufficient to cause a phenotypic change in *Foxa3^Hep−/−^* mice. In addition, hepatic genes involved in lipogenesis, inflammation, or fibrogenesis were largely unchanged (Supplementary Figure S2A,B), consistent with the unchanged hepatic or plasma lipid levels or the development of MASLD.

### 2.3. Genetic Ablation of Hepatocyte Foxa3 Improves Masld/Mash in Hyperlipidemic Ldlr-Deficient Mice

Previous studies have shown that overexpression of human FOXA3 in the liver prevents the development of atherosclerosis in *Apoe*^−/−^ mice [7]. To determine whether loss of hepatocyte FOXA3 had the opposite effect on atherosclerosis development, we i.v. injected *Foxa3^fl/fl^* mice with AAV8-D377Y-mPCSK9 [13,14] together with AAV8-TBG-Null or AAV8-TBG-Cre to generate *Ldlr*-deficient mice with or without *Foxa3* ablation in hepatocytes. The mice were then fed a Western diet for 16 weeks.

**Figure 3:**
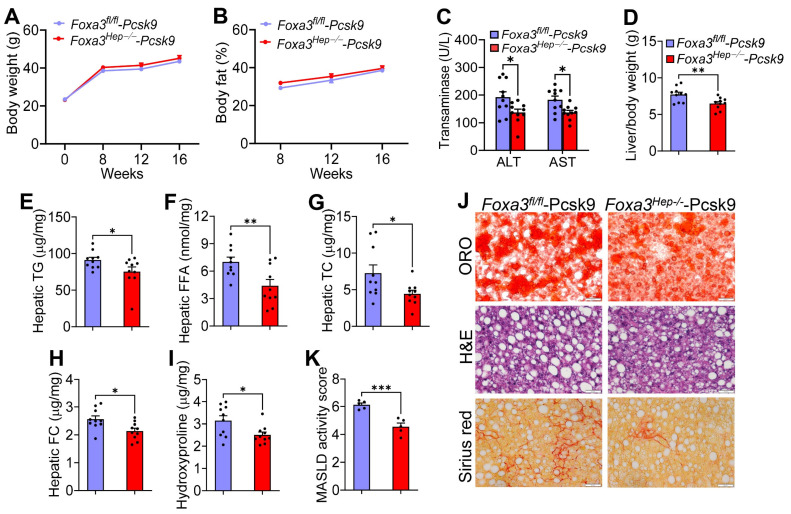
**Genetic loss of hepatocyte FOXA3 in *Ldlr*-deficient mice improves Western diet-induced steatohepatitis. ***Foxa3^fl/fl^-Pcsk9* mice and *Foxa3^Hep−/−^-Pcsk9* mice were fed a Western diet for 16 weeks (n = 10). (**A**) Body weight. (**B**) Body fat content (%). (**C**) Plasma ALT and AST levels. (**D**) Liver-to-body weight ratio. (**E**) Hepatic TG levels. (**F**) Hepatic FFA levels. (**G**) Hepatic TC levels. (**H**) Hepatic FC levels. (**I**) Hepatic hydroxyproline levels. (**J**) Histological staining of liver sections with oil red O (ORO), hematoxylin/eosin (**H**,**E**), or picrosirius red. (**K**) MASLD activity score. Scale bars in (**J**): 50 μm. Data are expressed as mean ± SEM. * *p* < 0.05, ** *p* < 0.01, *** *p* < 0.001.

Compared to *Foxa3^fl/fl^* mice expressing gain-of-function mutant PCSK9 (i.e., *Foxa3^fl/fl^-Pcsk9* mice), *Foxa3^Hep−/−^* mice expressing gain-of-function mutant PCSK9 (i.e., *Foxa3^Hep−/−^*-*Pcsk9* mice) had similar levels of body weight gain (Figure 3A) or body fat content (Figure 3B). Unexpectedly, *Foxa3^Hep−/−^*-*Pcsk9* mice had reduced plasma ALT and AST levels (Figure 3C) and liver-to-body weight ratio (Figure 3D). *Foxa3^Hep−/−^*-*Pcsk9* mice also had reduced hepatic levels of TG (Figure 3E), FFA (Figure 3F), TC (Figure 3G), FC (Figure 3H), and hydroxyproline (Figure 3I), as well as reduced MASLD activity score (Figure 3K). Histological staining by oil red O (ORO), H&E, or picrosirius red also confirmed that *Foxa3^Hep−/−^*-*Pcsk9* mice had decreased lipid accumulation and fibrosis in the liver (Figure 3J). Thus, the data of Figure 3 demonstrate that loss of hepatocyte FOXA3 ameliorates Western diet-induced MASLD/MASH in hyperlipidemic *Ldlr*-deficient mice.

### 2.4. Loss of Hepatocyte Foxa3 Prevents the Development of Atherosclerosis in Hyperlipidemic Ldlr-Deficient Mice

**Figure 4:**
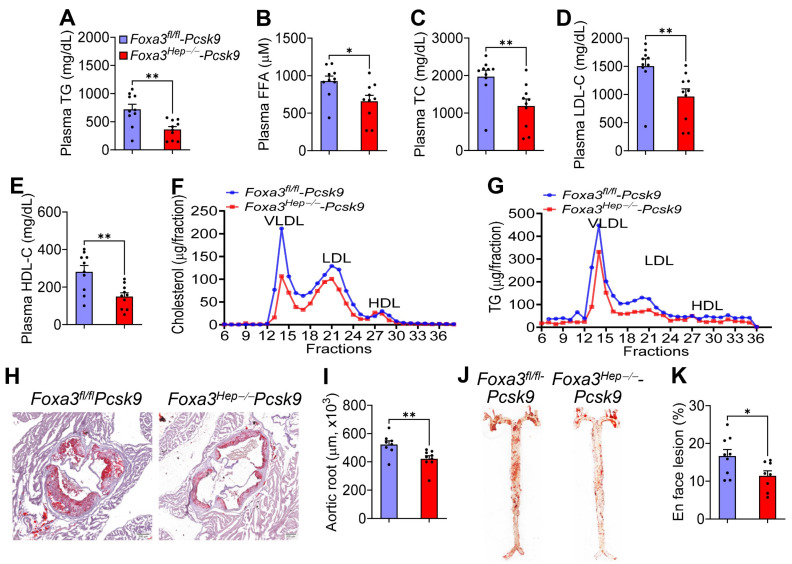
**Genetic loss of hepatocyte FOXA3 in *Ldlr*-deficient mice improves Western diet-induced hyperlipidemia and atherosclerosis. ***Foxa3^fl/fl^-Pcsk9* mice and *Foxa3^Hep−/−^-Pcsk9* mice were fed a Western diet for 16 weeks (n = 10). (**A**) Plasma TG levels. (**B**) Plasma FFA levels. (**C**) Plasma TC levels. (**D**) Plasma LDL-C levels. (**E**) Plasma HDL-C levels. (**F,G**) Analysis of plasma cholesterol (**F**) or triglyceride (TG) (**G**) lipoprotein profiles. (**H**,**I**) Aortic roots were stained with oil red O (**H**) and lesion areas quantified (**I**). (**J**,**K**) En face aortas were stained by oil red O (**J**), and lesion areas (%) quantified (**K**). Scale bars in (**H**): 200 μm. Data are expressed as mean ± SEM. * *p* < 0.05, ** *p* < 0.01, **** *p* < 0.0001.

In addition to the amelioration of MASLD/MASH, loss of hepatocyte FOXA3 also markedly reduced plasma levels of TG by 50% (Figure 4A), FFA by 29% (Figure 4B), TC by 40% (Figure 4C), LDL-C by 36% (Figure 4D), and HDL-C by 47% (Figure 4E). Analysis of plasma lipoprotein profile by fast protein liquid chromatography (FPLC) confirmed that *Foxa3^Hep−/−^*-*Pcsk9* mice had reduced VLDL-C and LDL-C (Figure 4F) as well as reduced VLDL-TG and LDL-TG (Figure 4G). Consistent with the changes in plasma lipoprotein levels, loss of hepatocyte *Foxa3* reduced atherosclerotic lesions by 19.4% in the aortic roots (Figure 4H,I) and by 32% in the *en face* aortas (Figure 4J,K). These data indicate that loss of hepatocyte FOXA3 prevents the development of atherosclerosis in hyperlipidemic *Ldlr*-deficient mice.

### 2.5. Loss of Hepatocyte Foxa3 Inhibits Lipogenic, Pro-Inflammatory, and Fibrogenic Genes in Hyperlipidemic Ldlr-Deficient Mice

**Figure 5:**
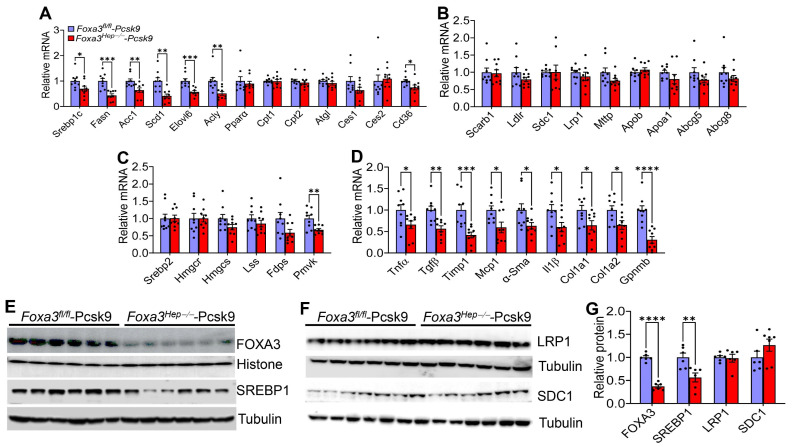
**Genetic loss of hepatocyte FOXA3 in *Ldlr*-deficient mice reduces Western diet-induced expression of lipogenic, proinflammatory, or fibrogenic genes. ***Foxa3^fl/fl^-Pcsk9* mice and *Foxa3^Hep−/−^-Pcsk9* mice were fed a Western diet for 16 weeks (n = 10). (**A**–**D**) Hepatic mRNA levels. (**E**–**G**) Hepatic protein levels were determined by Western blotting (**E**,**F**) and quantified (**G**). Data are expressed as mean ± SEM. * *p* < 0.05, ** *p* < 0.01, *** *p* < 0.001, **** *p* < 0.0001.

In the liver of *Foxa3^Hep−/−^*-*Pcsk9* mice, genes involved in lipogenesis or fatty acid uptake were significantly reduced, including sterol regulatory element-binding protein 1c (*Srebp1c*), fatty acid synthase (*Fasn*), acetyl-CoA carboxylase 1 (*Acc1*), stearoyl-CoA desaturase 1 (*Scd1*), elongation of very long chain fatty acid protein 6 (*Elov6*), ATP citrate lyase (*Acly*), and cluster of differentiation 36 (*Cd36*) (Figure 5A). There were no changes in genes involved in fatty acid oxidation [peroxisome proliferator-activated receptor alpha (*Ppara*), carnitine palmitoyltransferase 1 (*Cpt1*), *Cpt2*], lipolysis [adipose triglyceride lipase (*Atgl*), carboxylesterase 1(*Ces1*), *Ces2*], lipoprotein uptake [scavenger receptor group B type 1 (*Scarb1*), *Ldlr*, syndecan 1 (*Sdc1*), LDLR-related protein 1 (*Lrp1*)], VLDL secretion [microsomal triglyceride transfer protein (*Mttp*), apolipoprotein b (*Apob*)], cholesterol excretion [ATP-binding cassette subfamily G member 5 (*Abcg5*), *Abcg8*) or cholesterol synthesis [*Srebp2*, HMG-CoA reductase (*Hmgcr*), HMG-CoA synthase (*Hmgcs*), lanosterol synthase (*Lss*), farnesyl diphosphate synthase (*Fdps*)] except for a reduction of phosphomevalonate kinase (*Pmvk*) (Figure 5B, C). Most strikingly, many pro-inflammatory or fibrogenic genes were also significantly reduced, including Tumor necrosis factor alpha (*Tnfa*), transforming growth factor beta (*Tgfb*), tissue inhibitor of metalloproteinase 1 (*Timp1*), monocyte chemoattractant protein 1 (*Mcp1*), alpha-smooth muscle actin (*a-Sma*), interleukin 1 beta (*Il1b*), collagen type 1 alpha 1 chain (*Col1a1*), *Col1a2*, or glycoprotein non-metastatic melanoma protein B (*Gpnmb*) (Figure 5D).

Western blot assays showed that hepatic FOXA3 or SREBP1 protein levels were significantly reduced, whereas there was no change in hepatic LRP1 or SDC1 protein levels (Figure 5E–G). Taken together, the data in Figure 5 suggest that loss of hepatocyte FOXA3 in hyperlipidemic *Ldlr*-deficient mice lowers hepatic and plasma TG levels, likely by inhibiting lipogenic genes and fatty acid uptake, and prevents MASH development, likely by inhibiting proinflammatory and fibrogenic genes.

### 2.6. Loss of Hepatocyte Foxa3 Lowers Cholic Acid Levels and Reduces Bile Acid Hydrophobicity in Hyperlipidemic Ldlr-Deficient Mice

**Figure 6:**
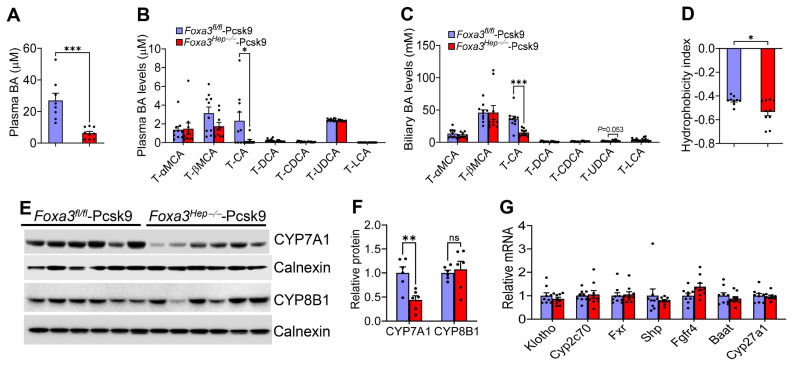
**Genetic loss of hepatocyte FOXA3 in Western diet-fed *Ldlr*-deficient mice reduces cholic acid levels and bile acid hydroxybicity. ***Foxa3^fl/fl^-Pcsk9* mice and *Foxa3^Hep−/−^-Pcsk9* mice were fed a Western diet for 16 weeks (n = 10). (**A**) Plasma bile acid (BA) levels. (**B**) Plasma BA composition. (**C**) Biliary BA composition. (**D**) BA hydrophobicity index. (**E**,**F**) Hepatic protein levels. (**G**) Hepatic mRNA levels. T-αMCA, tauro-α-muricholic acid; T-βMCA, tauro-β-muricholic acid; T-CA, taurocholic acid; T-DCA, taurodeoxycholic acid; T-CDCA, taurochenodeoxycholic acid; T-UDCA, tauroursodeoxycholic acid; T-LCA, taurolithocholic acid. Data are expressed as mean ± SEM. ns, not significant. * *p* < 0.05, ** *p* < 0.01, *** *p* < 0.001.

Bile acids are endocrine molecules that play an important role in regulating lipid absorption and cholesterol metabolism. In hyperlipidemic *Ldlr*-deficient mice, loss of hepatocyte FOXA3 reduced plasma bile acid levels by 77% (Figure 6A). Analysis of bile acid composition by LC-MS/MS showed that loss of hepatocyte FOXA3 reduced taura-cholic acid levels by 92% in the plasma (Figure 6B) and by 58% in the bile (Figure 6C). As a result, loss of hepatocyte FOXA3 significantly reduced the bile acid hydrophobicity index (Figure 6D). Interestingly, loss of hepatocyte FOXA3 significantly reduced hepatic CYP7A1 protein levels, but did not affect hepatic CYP8B1 protein levels (Figure 6E,F), or other genes involved in bile acid metabolism, including *Klotho*, *Cyp2c70*, *Fxr*, *Shp*, *Fgf4*, *Baat*, or *Cyp27a1* (Figure 6G). Given that cholic acid is very efficient in promoting lipid absorption, the markedly reduced cholic acid levels may, in part, account for the reduced hepatic and plasma lipid levels in *Foxa3^Hep−/−^*-*Pcsk9* mice.

## 3. Discussion

Hepatic FOXA3 is reduced in MASH patients and obese mice [7]. Overexpression of human FOXA3 in the liver is shown to protect against diet-induced obesity, MASH, and atherosclerosis [7,8]. However, no studies have investigated whether genetic inactivation of hepatocyte FOXA3 affects the development of MASH or atherosclerosis. In contrast to our hypothesis, genetic inactivation of hepatocyte FOXA3 does not affect the development of MASLD/MASH in C57BL/6 mice but ameliorates MASLD/MASH and atherosclerosis in hyperlipidemic *Ldlr*-deficient mice. This unexpected finding suggests that loss of hepatocyte FOXA3 may benefit metabolic homeostasis under severely hyperlipidemic conditions. It remains interesting to explore why this happens only in severely, but not mildly, hyperlipidemic mice.

*Foxa3^Hep−/−^*-*Pcsk9* mice are resistant to Western diet-induced MASLD/MASH and atherosclerosis, likely resulting from (1) reduced lipogenesis in the liver; (2) reduced hepatic fatty acid uptake due to decreased CD36 expression; (3) reduced hepatic inflammation and fibrogenesis; and (4) markedly reduced cholic acid levels and bile acid hydrophobicity. Previous studies have shown that reduced cholic acid levels or bile acid hydrophobicity reduce intestinal fat absorption and protect against the development of MASLD and atherosclerosis [15,16,17,18]. Thus, the markedly reduced cholic acid levels may play a key role in lowering plasma and hepatic lipid levels in *Foxa3^Hep−/−^*-*Pcsk9* mice. We speculate that under severely hyperlipidemic conditions, loss of hepatocyte FOXA3 in *Ldlr*-deficient mice may alter gut microbiota composition, which, in turn, affects bile acid metabolism, leading to a more hydrophilic bile acid pool, reduced fat absorption, and improved lipid homeostasis.

We previously showed that overexpression of human FOXA3 in the liver increases plasma bile acid levels by inhibiting NCTP and OATP expression, thereby increasing energy expenditure and reducing diet-induced obesity via activating TGR5 [8]. Overexpression of human FOXA3 also reduced hepatic CYP8B1 expression but slightly induced hepatic CYP7A1 expression [8]. In the current study, we show that genetic loss of hepatocyte FOXA3 in C57BL/6 mice results in a modest reduction in hepatic CYP7A1 expression and in plasma bile acid levels, an induction of hepatic NTCP expression, and no change in obesity, energy expenditure, or hepatic CYP8B1, CYP27A1, or CYP7B1 expression. CYP8B1 catalyzes the synthesis of cholic acid, whereas CYP27A1 and CYP7B1 catalyze bile acid synthesis in the alternative pathway. Thus, the reduction in plasma bile acid levels may result from reduced CYP7A1 expression and increased hepatic NTCP expression. Collectively, the data from loss-of-function studies in C57BL/6 mice only partially support the findings from gain-of-function studies. We speculate that human and mouse FOXA3 may have overlapping yet distinct functions. In addition, gain- or loss-of-function studies may induce distinct compensatory effects that affect overall phenotypic observations.

Previously, Liu et al. showed that global *Foxa3*^−/−^ mice have reduced hepatic TG levels after acute treatment with tunicamycin, an ER stress inducer [5]. They also showed that genetic or siRNA-mediated global inactivation of FOXA3 reduces high-fat diet-induced steatosis [5]. Since the inactivation of FOXA3 in hepatocytes does not affect the development of MASLD, the changes observed by Liu et al. [5] likely result from the inhibition of extra-hepatic FOXA3, as FOXA3 is also expressed in other organs, such as the intestine. In addition, differences in gut microbiota composition across facilities may contribute to the observed discrepancies.

In summary, genetic inactivation of hepatocyte FOXA3 does not affect the development of MASLD/MASH in Western diet-fed C57BL/6 mice but prevents Western diet-induced MASLD/MASH and atherosclerosis in hyperlipidemic *Ldlr*-deficient mice. Further studies are needed to determine the underlying mechanisms more precisely.

## 4. Methods and Materials

### 4.1. Mice and Diets

C57BL/6J mice (stock #000664) were purchased from The Jackson Laboratory (Bar Harbor, ME, USA). Floxed Foxa3 mice, with loxP sites flanking exon 2, were generated by Biocytogen on a C57BL/6 background. Homozygous Foxa3^fl/fl^ mice were generated by crossbreeding Foxa3^fl+/−^ mice. The primer sequences for genotyping are 5′-CATGGCCTTGAAGGTATGGAAGG-3′ (F) and 5′-GTGGGCACAGGATTCACTGGAGA-3′ (WT: 372 bp; Flox: 510 bp). Hepatocyte-specific Foxa3^−/−^ mice were generated by i.v. injection of Foxa3^fl/fl^ mice with AAV8-TBG-Cre. All mice were housed in a temperature- and humidity-controlled room with a 12-h light/12-h dark cycle and free access to water and food. The Western diet (42% kcal from fat/0.2% cholesterol) was purchased from Envigo (Cat # TD.88137). The high-fat/cholesterol/fructose (HFCF) diet containing 21% fats, 0.21% cholesterol, 15% fructose, and 35% sucrose was purchased from Research Diets (Cat # D16051004). For the feeding studies, 8- to 10-week-old male mice were randomly allocated and fed either a Western diet or an HFCF diet for 16 weeks. Unless otherwise stated, mice were fasted for 5–6 h before euthanasia. All animal experiments were approved by the Institutional Animal Care and Use Committee at Northeast Ohio Medical University (Protocol ID: 22-12-349, 7 April 2023).

### 4.2. Adeno-Associated Viruses (AAVs)

AAV8-TBG-Null (control), AAV8-TBG-Cre, and AAV8-D377Y-mPCSK9 [13,14] were produced and titrated by Vector Biolabs. Mice were intravenously injected with 2 × 10^11^ genome copies (GC) of AAVs before being placed on a special diet.

### 4.3. Quantitative Real-Time PCR

Total RNA was isolated using TRIzol Reagent (ThermoFisher, Waltham, MA, USA). mRNA levels were determined by quantitative reverse-transcription polymerase chain reaction (qRT-PCR) on a 7500 real-time PCR system (Applied Biosystems, Foster City, CA, USA) using SYBR Green Supermix (ThermoFisher, Waltham, MA, USA). Relative mRNA levels were quantified using the 2^^−∆∆Ct^ method and normalized to *36b4*.

### 4.4. Western Blot Assays

Western blot assays were performed using whole liver lysate extracts or microsomes of the liver samples as described [13]. The gel band intensity was quantified using ImageJ 1.54g and normalized to loading controls. Antibodies against FOXA3 (cat # sc-74424), histone (cat # sc-393358), and SDC1 (cat # sc-12765) were purchased from Santa Cruz Biotechnology. Antibodies against CYP7A1 (cat # TA351400) and CYP8B1 (cat # TA313734) were purchased from Origene. Antibodies against LRP1 (cat. # ab92544; 1:1000 dilution) or tubulin (cat. # ab4074; 1:2000 dilution) were purchased from Abcam. Antibodies against SREBP-1 (cat # NB600-582; 1:1000 dilution) or calnexin (cat # NB100-1965; 1:1000 dilution) were purchased from Novus Biologicals, Centennial, CO, USA.

### 4.5. Plasma and Liver Biochemistry

Plasma alanine aminotransferase (ALT), aspartate aminotransferase (AST), triglyceride (TG), and total cholesterol (TC) levels were determined using Infinity reagents from ThermoFisher (Waltham, MA, USA). Plasma HDL cholesterol (HDL-C) levels were measured using the HDL-Cholesterol Liquid Reagent (SB-0599-020, Stanbio Laboratory, Boerne, TX, USA). Total bile acids were measured using the Diazyme Total Bile Acid Test kit (cat # DZ042A-K01, Diazyme Laboratories Inc, San Diego, CA, USA). Plasma LDL cholesterol (LDL-C) levels were measured using Diazyme LDL-cholesterol assay (DZ128A-KY1, Diazyme Laboratories Inc). Plasma lipoproteins were separated by the Biologic DuoFlow QuadTec 10 System (Bio-Rad, Hercules, CA, USA) as described [19], and cholesterol or triglyceride levels in each fraction were quantified. To measure hepatic lipid levels, approximately 100 mg of liver tissue was homogenized in methanol, and lipids were extracted using chloroform/methanol (2:1 *v*/*v*) as described [20]. Hepatic TG or total cholesterol levels were quantified using Infinity reagents (ThermoFisher, Waltham, MA, USA). Hepatic free fatty acids (FFAs) and free cholesterol (FC) were determined using kits from Wako Chemicals USA (Richmond, VA, USA).

### 4.6. Bile Acid Levels, Bile Acid Pool Size, and Bile Acid Composition

Bile acids in the liver, gall bladder, and intestine were extracted using ethanol [21]. Total bile acids in the liver, gallbladder, and intestine were determined to calculate the total bile acid pool size. The bile acid composition in the plasma or bile was determined by LC-MS/MS [8]. The bile acid hydrophobicity index was calculated as described by Heuman [22].

### 4.7. Oil Red O (Oro), Hematoxylin and Eosin (H&e) Staining, and Picrosirius Red Staining

Liver tissues were fixed in 10% formalin and then embedded in OCT or paraffin. The liver sections were stained with ORO, H&E, or picrosirius red. Hepatic neutral lipid accumulation and morphology were determined using images acquired by an Olympus microscope.

### 4.8. Body Composition and Energy Expenditure

EchoMRI-700 (EchoMRI LLC, Houston, TX, USA) was used to measure whole-body fat and lean mass. The comprehensive laboratory animal monitoring system (CLAMS) was used to measure oxygen consumption and heat production as described [23,24]. The data were analyzed using the web-based CalR program [25].

### 4.9. Glucose Tolerance Test

Mice were fasted for 16 h and then injected i.p. with 2 g/kg body weight of glucose. Plasma glucose levels were determined at 0, 30, 60, 90, or 120 min using a glucometer.

### 4.10. Atherosclerotic Lesion Quantification

The whole aorta, including the ascending, thoracic, and abdominal segments, was isolated and cleaned under a microscope. *En face* Aortas were stained with Oil Red O. Aortic roots were sectioned at the location of the aortic valve and stained with Oil Red O. The atherosclerotic plaque size was determined using ImagePro software 10.

### 4.11. Statistical Analysis

All data were expressed as mean ± SEM. Statistical significance was analyzed by Prism (GraphPad, San Diego, CA, USA) using an unpaired Student *t*-test or ANOVA for multiple comparisons. Differences were considered statistically significant at *p* < 0.05.

## 5. Conclusions

This study provides the first evidence showing that loss of hepatocyte FOXA3 has no impact on Western diet-induced lipid homeostasis or MASH development in C57BL/6 mice. However, in *Ldlr*-deficient mice, loss of hepatocyte FOXA3 improves Western diet-induced MASH and atherosclerosis. The latter changes are associated with reduced expression of lipogenic, proinflammatory, and fibrogenic genes, as well as reduced bile acid hydrophobicity. Further studies are needed to address why the phenotypic changes induced by hepatocyte FOXA3 deficiency are observed only under hyperlipidemic conditions.

## Data Availability

The original contributions presented in this study are included in the article/Supplementary Material. Further inquiries can be directed to the corresponding author.

## Supplementary Materials

The following supporting information can be downloaded at: https://www.mdpi.com/article/10.3390/ijms27031468/s1.
