# Supplementary material for: Loss of Hepatocyte FOXA3 Improves MASH and Atherosclerosis in Hyperlipidemic Ldlr-Deficient Mice
Source: Int J Mol Sci. 2026 Feb 2;27(3):1468. doi: 10.3390/ijms27031468 (PMC12898518; doi:10.3390/ijms27031468)
Supplement: Supplementary file 1 [file ijms-27-01468-s001.zip › ijms-4113772-supplementary.pdf]

# Supplementary Information

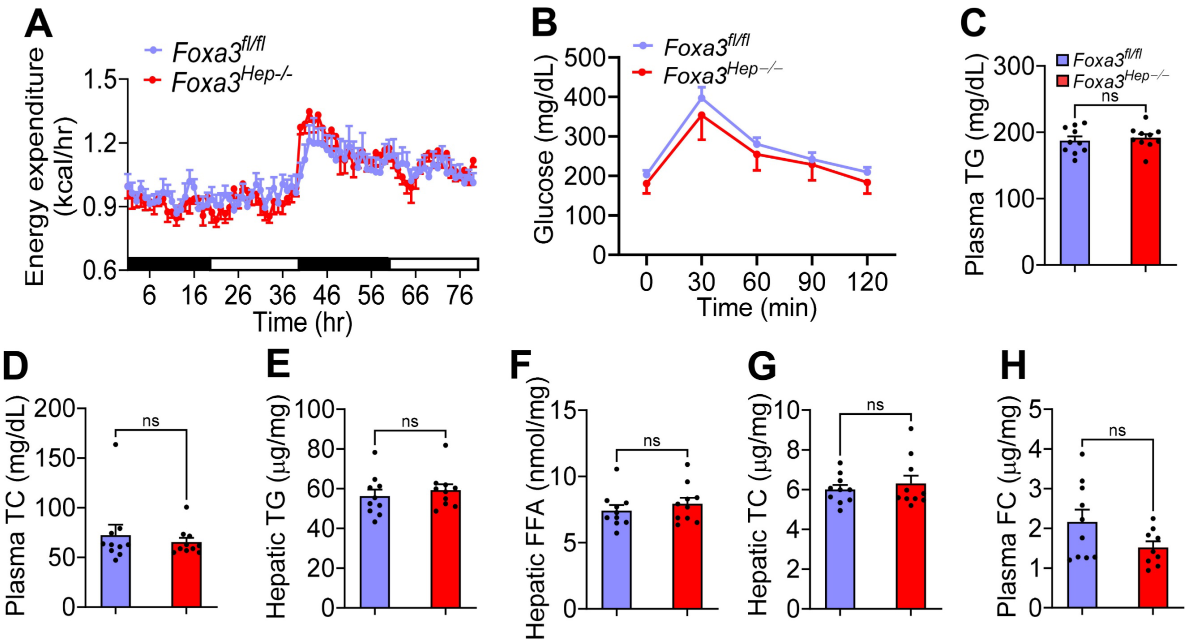


**Supplementary Figure 1. Genetic loss of hepatocyte FOXA3 in Western diet-fed C57BL/6 mice has no impact on lipid homeostasis or MASLD**

*Foxa3*$^{fl/fl}$ mice and *Foxa3*$^{Hep-/-}$ mice were fed a Western diet for 16 weeks (n=9-10 per group). (**A**) Energy expenditure. (**B**) Glucose tolerance test. (**C**) Plasma TG levels. (**D**) Plasma TC levels. (**E**) Hepatic TG levels. (**F**) Hepatic FFA levels. (**G**) Hepatic TC levels. (**H**) Hepatic FC levels. Data are expressed as mean ± SEM. ns, not significant

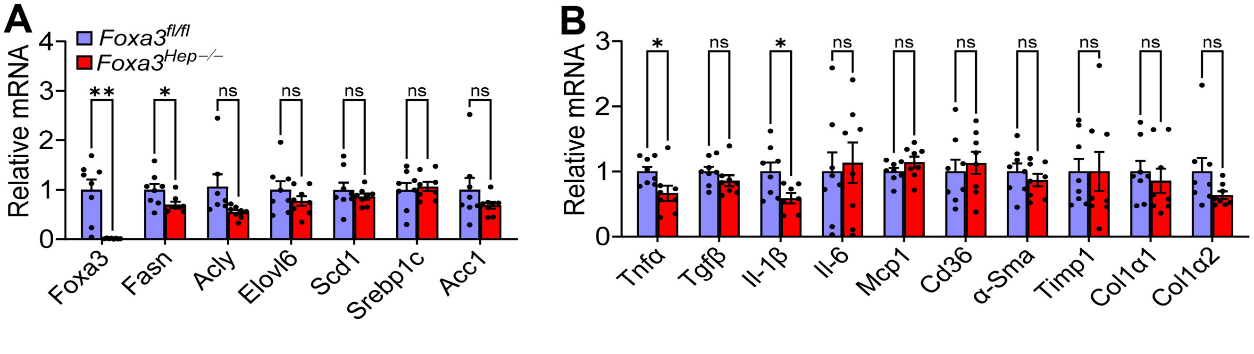


**Supplementary Figure 2. Hepatic mRNA levels in HFCF diet-fed mice**

*Foxa3*$^{fl/fl}$ mice and *Foxa3*$^{Hep-/-}$ mice were fed an HFCF diet for 16 weeks (n=8 per group). (**A-B**) Hepatic mRNA levels were determined. Data are expressed as mean ± SEM. ns, not significant. *$P$<0.05, **$P$<0.01
